# Association of the triglyceride-glucose index and its derived indices with carotid artery plaques in postmenopausal women: a cross-sectional study

**DOI:** 10.1186/s13104-025-07597-2

**Published:** 2025-12-24

**Authors:** Xiaoqin Chen, Xueru Ding, Min Liang, Li Li, Xia Dong, Xiang Zhao

**Affiliations:** 1 Coronary Care Unit, Heart Center, The First Affiliated Hospital of Xinjiang Medical University, Urumqi, Xinjiang China; 2 Second Department of Coronary Heart Disease, The First Affiliated Hospital of Xinjiang Medical University, Urumqi, Xinjiang China

**Keywords:** Postmenopausal women, Triglyceride glucose index, Carotid artery plaque, Cross-sectional study

## Abstract

**Objective:**

This study aimed to compare the associations of the triglyceride-glucose (TyG) index and its obesity-combined derivatives (TyG-BMI, TyG-WC, TyG-WHtR) with carotid artery plaques in a cohort of postmenopausal women.

**Results:**

Among 435 postmenopausal women studied, the prevalence of carotid plaques was 36.6% (*n* = 159). Compared to the non-plaque group, women with plaques were older and had higher triglyceride levels and TyG index (all *P* < 0.01). In multivariable logistic regression analysis, after full adjustment for confounders including age, LDL-C, and cardiometabolic histories, only the TyG index maintained an independent association with plaque presence (OR = 1.471, 95% CI 1.044–2.073, *P* = 0.027). In contrast, none of the derivatives (TyG-BMI, TyG-WC, TyG-WHtR) showed significant associations. The TyG index demonstrated a modest predictive value for plaques (AUC = 0.593, *P* = 0.001), which was superior to its derivatives.

## Introduction

Cardiovascular diseases (CVD) represent the leading cause of mortality and disability among women globally, with a marked surge in risk following menopause [1]. Evidence indicates that the incidence of major adverse cardiovascular events, such as coronary heart disease and stroke, is significantly higher in postmenopausal women compared to their premenopausal counterparts or age-matched men [2]. Furthermore, the timing of menopause is closely linked to CVD risk, with premature or early onset menopause associated with a substantially elevated risk [3]. This phenomenon cannot be fully attributed to an increase in traditional cardiovascular risk factors alone, suggesting the involvement of distinct sex-specific pathophysiological mechanisms.

The cessation of ovarian function and the consequent sharp decline in endogenous estrogen levels are pivotal drivers of this risk transition. Estrogen confers multifaceted protective effects on the cardiovascular system, including lipid metabolism regulation, enhancement of endothelial function, and suppression of inflammatory and oxidative stress pathways [4–7]. The withdrawal of this endogenous protection predisposes women to a cluster of metabolic disturbances, such as central obesity, insulin resistance (IR), and dyslipidemia—characterized by elevated triglycerides and reduced high-density lipoprotein cholesterol (HDL-C) [8, 9]. Consequently, the elevated cardiovascular risk in postmenopausal women can be largely ascribed to the deterioration of their unique metabolic risk profile [10].

Within this context, the early identification of subclinical atherosclerosis is paramount for risk assessment and primary prevention. Carotid atherosclerotic plaques serve as a reliable “window” to systemic atherosclerosis. The detection of carotid intima-media thickness and plaques has been validated as an effective means to evaluate individual cardiovascular risk and guide preventive strategies [11, 12]. High-frequency ultrasonography for carotid plaque assessment is a simple, non-invasive, and highly reproducible technique, now established as a key tool in cardiovascular risk evaluation. Investigating modifiable risk factors closely linked to carotid plaques in postmenopausal women holds significant clinical relevance.

The distinct metabolic landscape of postmenopause, particularly the rise in insulin resistance (IR), necessitates a re-evaluation. Insulin resistance, often estimated by the triglyceride-glucose (TyG) index, is a central driver of atherosclerosis [13]. It has demonstrated superior performance over conventional single lipid or glycemic metrics in predicting incident diabetes, the severity of coronary artery disease, and future cardiovascular events [14–16]. To enhance predictive power, several obesity-integrated derivatives of the TyG index, such as TyG-BMI, TyG-waist circumference (TyG-WC), and TyG-waist-to-height ratio (TyG-WHtR), have been developed and validated primarily in general or male-dominated cohorts [17–19].

Despite the established link between the TyG index and atherosclerosis, its behavior specifically in postmenopausal women—and crucially, the comparative utility of its widely studied derivatives—remains unclear. Existing research on this relationship has predominantly been conducted in general populations or with significant inclusion of male participants, whose metabolic and body composition profiles differ substantially from those of postmenopausal females [20, 21]. This gap is critical because risk assessment tools derived from one demographic may not translate directly to another with distinct pathophysiology. Therefore, this study aimed to investigate and compare the associations of the TyG index and its key derivatives (TyG-BMI, TyG-WC, TyG-WHtR) with the presence of carotid artery plaques exclusively in a cohort of postmenopausal women.

### Study design and population

This was a single-center, retrospective, cross-sectional study. The study population consisted of postmenopausal women consecutively admitted to the Department of Cardiology at the First Affiliated Hospital of Xinjiang Medical University between January 2020 and December 2023, with a primary complaint of chest pain or precordial discomfort. Eligible participants met all the following criteria: (1) natural menopause (amenorrhea for ≥ 12 months); (2) underwent carotid ultrasound during hospitalization; (3) had complete baseline clinical and laboratory data. Exclusion criteria were: (1) history of carotid endarterectomy or stenting; (2) severe hepatic or renal dysfunction, malignancy, thyroid disorders, or other systemic diseases significantly affecting metabolism; (3) missing key variables (e.g., menarche age, menopause age, lipid profiles, blood glucose). Patients with any missing data for the key variables required for analysis (including TyG index components, carotid ultrasound results, or listed confounders) were excluded from the initial cohort screening, resulting in a complete-case analysis. This study was approved by the Medical Ethics Committee of the First Affiliated Hospital of Xinjiang Medical University (Approval No.: 240104-02) on 8 January 2024. The requirement for informed consent was waived by the ethics committee due to the retrospective nature of the study and minimal risk to participants.

### Data collection and definitions

Two trained researchers independently extracted the following data by reviewing the electronic medical record system:

Demographic and clinical data: Age, smoking status, alcohol consumption, medical history (hypertension, diabetes, chronic angina pectoris, stroke), age at menarche, and age at menopause.

Anthropometric measurements: Height, weight, and waist circumference (WC). Body mass index (BMI) was calculated as weight (kg) divided by height squared (m²).

Laboratory parameters: Fasting venous blood samples were collected the morning after admission. Measured parameters included fasting blood glucose (FBG), triglycerides (TG), total cholesterol (TC), high-density lipoprotein cholesterol (HDL-C), and low-density lipoprotein cholesterol (LDL-C).

Calculation of TyG Index and Derived Indices:

$${\mathrm{TyG}}\;{\mathrm{index}}={\text{Ln }}\left[ {{\mathrm{TG}}\left( {{\mathrm{mg}}/{\mathrm{dL}}} \right) \times {\mathrm{FBG}}\left( {{\mathrm{mg}}/{\mathrm{dL}}} \right)/2} \right]$$

$${\mathrm{TyG-BMI}}={\mathrm{TyG}}\;{\mathrm{index}} \times {\mathrm{BMI}}$$

$${\mathrm{TyG-WC}}={\mathrm{TyG}}\;{\mathrm{index}} \times {\mathrm{WC}}\left( {{\mathrm{cm}}} \right)$$

$${\mathrm{TyG-WHtR}}={\mathrm{TyG}}\;{\mathrm{index}} \times \left[ {{\mathrm{WC}}\left( {{\mathrm{cm}}} \right)/{\mathrm{Height}}\left( {cm} \right)} \right]$$

### Assessment of carotid artery plaques

All carotid ultrasound examinations were performed by experienced sonographers using color Doppler ultrasound systems. The scanning protocol encompassed the bilateral common carotid arteries, carotid bifurcations, internal carotid arteries, innominate arteries, and subclavian arteries. A carotid plaque was defined as a focal structure protruding into the vessel lumen in any of the scanned segments, with an intima-media thickness (IMT) ≥ 1.5 mm or exceeding 50% of the IMT of the adjacent arterial wall. Based on this definition, patients were classified into “Plaque Group” and “Non-Plaque Group.” For patients with plaques, the specific location (common carotid, internal carotid, innominate, subclavian) of each plaque was recorded, and its length and maximal thickness were measured. For each patient, only the thickest plaque value was recorded per vascular site (e.g., left common carotid); if no plaque was present at a site, the plaque thickness was recorded as 0. This allowed for the calculation of the “maximal plaque thickness” per vascular site per patient and further computation of the average plaque burden across the entire plaque group for different locations (including patients without plaques at that site). The maximum plaque thickness (MPT) was defined as the greatest thickness among all plaques measured across all examined vessels, calculated solely from patients with plaques.

### Statistical analysis

All statistical analyses were conducted using IBM SPSS Statistics (Version 25.0). A two-sided P-value < 0.05 was considered statistically significant. Continuous variables were tested for normality using the Shapiro-Wilk test. Normally distributed data are presented as Mean ± Standard Deviation (SD) and compared using the independent samples t-test. Non-normally distributed data are presented as Median (Interquartile Range, IQR) and compared using the Mann-Whitney U test. Categorical variables are presented as numbers (percentages) and compared using the Chi-square test or Fisher’s exact test, as appropriate. To control for potential confounders, a series of hierarchically adjusted multivariable binary logistic regression models were constructed to assess the associations between the indices and carotid plaque presence. Results are expressed as Odds Ratios (OR) with 95% Confidence Intervals (CI). Multicollinearity among the independent variables was assessed using the Variance Inflation Factor (VIF). A VIF value exceeding 5 was considered indicative of significant multicollinearity. Model 1: Adjusted for age and menopause age. Model 2: Model 1+ additional adjustment for smoking, alcohol consumption, history of diabetes, and history of hypertension. Model 3: Model 2+ further adjustment for LDL-C level. The predictive performance of the various indices for carotid plaque presence was evaluated using Receiver Operating Characteristic (ROC) curve analysis, and the Area Under the Curve (AUC) was calculated.

## Results

### Baseline characteristics of the study population

The final analysis included 435 postmenopausal women with a mean age of 59.3 ± 8.3 years. Carotid plaques were detected by ultrasound in 159 patients (36.6%), constituting the Plaque Group, while the remaining 276 patients (63.4%) formed the Non-Plaque Group. A comparison of baseline characteristics between the two groups is detailed in Table 1. Participants in the Plaque Group were significantly older and had higher TG levels and TyG index values compared to those in the Non-Plaque Group (all *P* < 0.01). Additionally, a higher proportion of patients in the Plaque Group had a history of chronic angina pectoris (*P* = 0.026). No statistically significant differences were observed between the groups regarding age at menarche, age at menopause, BMI, WC, other lipid parameters (TC, HDL-C, LDL-C), FBG, or histories of diabetes and hypertension.

**Table 1:** Baseline characteristics of postmenopausal women stratified by carotid plaque status

Characteristic	Non-plaque group (*n* = 276)	Plaque group (*n* = 159)	*P*
Age, years	58 ± 8	61 ± 8	< 0.001
Menarche age, years	13 ± 2	14 ± 2	0.053
Menopause age, years	48 ± 4	48 ± 4	0.854
Smoking, n (%)	2 (0.7)	1 (0.6)	0.908
Alcohol consumption, n (%)	4 (1.4)	4 (2.5)	0.425
Diabetes, n (%)	44 (15.9)	33 (20.8)	0.205
Hypertension, n (%)	114 (41.3)	80 (50.3)	0.069
Chronic angina, n (%)	35 (12.7)	33 (20.8)	0.026
Stroke, n (%)	9 (3.3)	4 (2.5)	0.660
BMI, kg/m²	26.1 ± 4.5	26.1 ± 4.5	0.962
WC, cm	86 ± 6	85 ± 6	0.707
FBG, mg/dL	94 (85, 119)	95 (86, 165)	0.469
TG, mg/dL	117 (86, 164)	140 (103, 187)	< 0.001
TC, mg/dL	94 (85, 119)	95 (86, 125)	0.420
HDL-C, mg/dL	46 (38, 54)	45 (36, 52)	0.558
LDL-C, mg/dL	102 (81, 123)	102 (82, 128)	0.492
TyG Index	8.68 (8.51, 9.32)	8.84 (8.51, 9.32)	0.001
TyG-BMI	224.20 (194.49, 258.81)	227.76 (201.25, 262.41)	0.300
TyG-WC	744.70 (686.84, 806.69)	754.43 (707.04, 795.14)	0.116
TyG-WHtR	4.58 (4.28, 5.01)	4.72 (4.36, 5.10)	0.099

*BMI* Body mass index, *WC* Waist circumference, *FBG* Fasting blood glucose, *TG* Triglycerides, *TC* Total cholesterol, *HDL-C* High-density lipoprotein cholesterol, *LDL-C* Low-density lipoprotein cholesterol, *TyG* Triglyceride-glucose index, *TyG-BMI* TyG index × BMI, *TyG-WC* TyG index × Waist circumference, *TyG-WHtR* TyG index × Waist-to-height ratio

### Distribution and burden of carotid plaques

Among the 159 patients with plaques, a detailed analysis of plaque distribution and burden was performed (Table 2). Plaques were most frequently found in the internal carotid arteries (75.5%), followed by the innominate arteries (42.8%) and subclavian arteries (36.5%), with the common carotid arteries being the least involved (19.5%). Regarding plaque burden, the internal carotid arteries exhibited the greatest average maximal plaque thickness and area.

**Table 2:** Distribution and burden of carotid plaques in patients with plaques

Vessel	Patients with plaques, *n* (%)	Maximal plaque thickness (mm), Mean ± SD	Maximal plaque area (mm²), Mean ± SD
Common carotid artery	31 (19.5)	0.44 ± 0.94	6.44 ± 16.13
Internal carotid artery	120 (75.5)	1.70 ± 1.20	16.98 ± 15.62
Innominate artery	68 (42.8)	1.12 ± 1.56	11.21 ± 15.93
Subclavian artery	58 (36.5)	0.93 ± 1.32	10.11 ± 16.58

### Associations of TyG index and its derived indices with carotid plaques

**Fig. 1:**
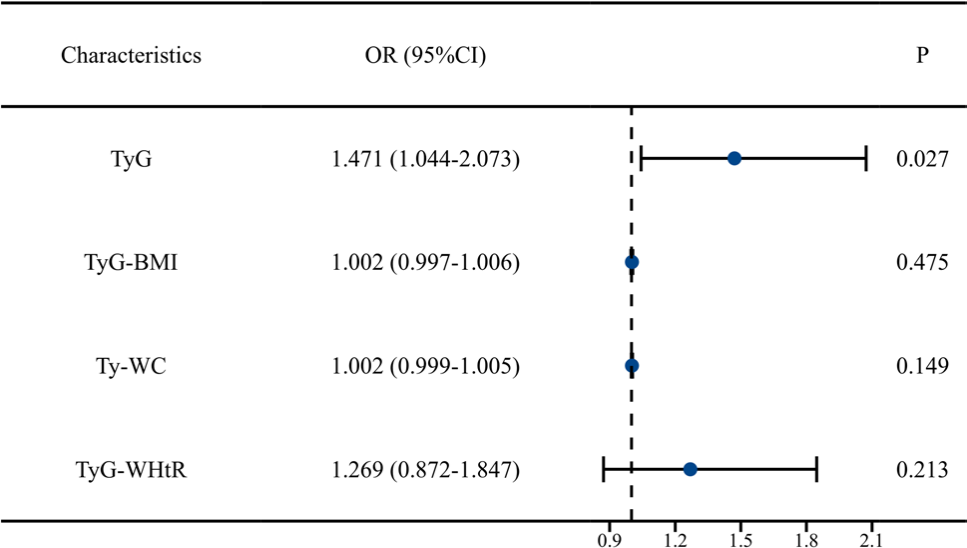
Forest plot of the association between various indices and carotid plaques in the fully adjusted model. The plot displays the odds ratios (OR) and 95% confidence intervals from the multivariable logistic regression Model 3 (adjusted for age, menopause age, smoking, alcohol consumption, history of diabetes, history of hypertension, and LDL-C)

Multivariable logistic regression analysis revealed (Table 3) that in the unadjusted model, the TyG index was significantly associated with plaque presence (OR = 1.597, 95% CI 1.178–2.165, *P* = 0.003). This association remained statistically significant after sequential adjustment for multiple confounders in Models 1–3 (fully adjusted OR in Model 3 = 1.471, 95% CI 1.044–2.073, *P* = 0.027). Conversely, none of the derived indices (TyG-BMI, TyG-WC, TyG-WHtR) showed a statistically significant association with carotid plaques in any of the adjusted models (all *P* > 0.05). A forest plot visually summarizing the key findings from the fully adjusted model (Model 3) is presented in Fig. 1. It clearly demonstrates that only the TyG index maintained a statistically significant association with carotid plaques after full adjustment for covariates. An assessment of multicollinearity was performed for the fully adjusted model (Model 3). The results indicated that all VIF values were well below the threshold of 5 (e.g., for the TyG index, VIF = 1.232), confirming the absence of significant multicollinearity among the covariates.

**Table 3:** Multivariate logistic regression analysis of TyG index and its derived indices with the presence of carotid plaques

	OR (95%CI)	*P*
TyG		
Unadjusted	1.597 (1.178–2.165)	0.003
Model1	1.506 (1.104–2.057)	0.010
Model2	1.524 (1.089–2.132)	0.014
Model3	1.471 (1.044–2.073)	0.027
TyG-BMI		
Unadjusted	1.002 (0.998–1.007)	0.282
Model1	1.003 (0.998–1.007)	0.229
Model2	1.002 (0.998–1.007)	0.362
Model3	1.002 (0.997–1.006)	0.475
TyG-WC		
Unadjusted	1.002 (1.000–1.005)	0.076
Model1	1.002 (1.000–1.005)	0.054
Model2	1.002 (1.000–1.005)	0.090
Model3	1.002 (0.999–1.005)	0.149
TyG-WHtR		
Unadjusted	1.326 (0.948–1.855)	0.099
Model1	1.348 (0.958–1.898)	0.087
Model2	1.323 (0.915–1.913)	0.137
Model3	1.269 (0.872–1.847)	0.213

*OR* Odds Ratio, *CI* Confidence Interval, *TyG* Triglyceride-glucose index, *TyG-BMI* TyG index × BMI, *TyG-WC* TyG index × waist circumference, *TyG-WHtR* TyG index × waist-to-height ratio

Model 1: Adjusted for age and menopause age. Model 2: Model 1 + additional adjustment for smoking, alcohol consumption, history of diabetes, and history of hypertension. Model 3: Model 2 + further adjustment for LDL-C level

### Predictive performance of different indices

**Fig. 2:**
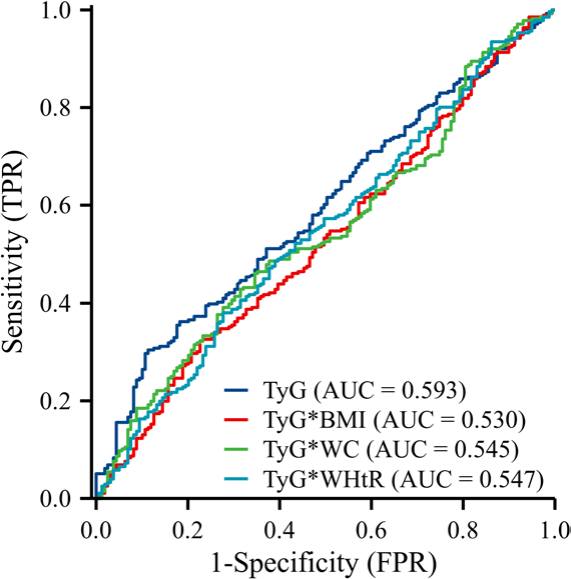
Receiver operating characteristic (ROC) curves for the TyG index and its derived indices

ROC curve analysis (Table 4) indicated that the TyG index possessed a modest but statistically significant predictive value for carotid plaque presence (AUC = 0.593, 95% CI 0.539–0.647, *P* = 0.001). In contrast, all TyG-derived indices (TyG-BMI, TyG-WC, TyG-WHtR) and traditional single parameters (BMI, WC, LDL-C) yielded AUC values that were not statistically significant and were relatively low, indicating limited discriminatory ability. For a visual comparison, the ROC curves for the TyG index and its key derivatives are depicted in Fig. 2, confirming the superior, albeit modest, discriminatory capacity of the TyG index alone.

**Table 4:** Predictive performance of various indices for the presence of carotid plaques: ROC curve analysis

	AUC (95%CI)	*P*
TyG	0.593 (0.539–0.647)	0.001
TyG-BMI	0.530 (0.474–0.586)	0.300
TyG-WC	0.545 (0.490–0.601)	0.116
TyG-WHtR	0.547 (0.492–0.603)	0.099
BMI	0.499 (0.442–0.555)	0.962
WC	0.489 (0.433–0.546)	0.707
LDL-C	0.520 (0.463–0.576)	0.492

*AUC* Area Under the Curve, *CI* Confidence Interval, *TyG* Triglyceride-glucose index, *TyG-BMI* TyG index × BMI, *TyG-WC* TyG index × waist circumference, *TyG-WHtR* TyG index × waist-to-height ratio, *BMI* Body mass index, *WC* waist circumference, *LDL-C* Low-density lipoprotein cholesterol

### Subgroup analysis

To assess the robustness of the association between the TyG index and plaques, stratified analyses were conducted based on diabetic status, obesity status (using BMI = 28 kg/m² as cutoff), and median menopause age (Table 5). Although the point estimates (OR) for the association between the TyG index and plaques were consistently greater than 1 across all subgroups, aligning with the primary analysis, the associations within individual subgroups did not reach statistical significance (all *P* > 0.05). However, tests for interaction were non-significant (all P-interaction > 0.05), indicating no statistically significant heterogeneity in the strength of the association between the TyG index and carotid plaques across patient subgroups defined by these characteristics.

**Table 5:** Subgroup analysis of the association between TyG index and carotid plaques

Subgroup	OR (95% CI)	*P*-value	*P* for interaction
Diabetes			0.961
Yes (*n* = 77)	1.529 (0.776–3.011)	0.22	
No (*n* = 358)	1.390 (0.917–2.107)	0.121	
Obesity (BMI ≥ 28)			0.871
Yes (*n* = 138)	1.847 (0.994–3.434)	0.052	
No (*n* = 297)	1.347 (0.878–2.066)	0.172	
Menopause age (median = 49)			0.995
≥ 49 years (*n* = 224)	1.305 (0.826–2.062)	0.253	
s< 49 years (*n* = 211)	1.713 (0.989–2.966)	0.055	

*OR* Odds Ratio, *CI* Confidence Interval, *BMI* Body mass index

Analyses are based on the fully adjusted model (Model 3). P for interaction was derived from the likelihood ratio test

## Discussion

This single-center, retrospective, cross-sectional study investigated the relationships between the TyG index, its derivatives, and carotid artery plaques in postmenopausal women. The principal findings are as follows: (1) The prevalence of carotid plaques was 36.6% in the cohort of 435 postmenopausal women. (2) Patients with plaques were older, had higher triglyceride levels and TyG index values, and a greater prevalence of chronic angina pectoris. (3) In multivariable logistic regression analyses, the TyG index was the sole parameter independently associated with plaque presence, whereas its derivatives incorporating obesity parameters (TyG-BMI, TyG-WC, TyG-WHtR) showed no significant association. (4) ROC curve analysis indicated a modest, yet significant, discriminatory ability of the TyG index for plaque presence (AUC = 0.593, *P* = 0.001), though its predictive power was limited. (5) Subgroup analyses demonstrated consistent directional trends (OR > 1) for the TyG-plaque association across various strata, albeit without reaching statistical significance within subgroups, and interaction tests revealed no significant effect modification.

The primary findings align with the established theoretical framework linking exacerbated metabolic disturbances to increased cardiovascular risk in postmenopausal women. The dramatic decline in endogenous estrogen levels following menopause is a pivotal trigger for a significant shift in cardiovascular risk trajectory. This hormonal change not only promotes dyslipidemia (e.g., high TG, low HDL-C) but is also a central driver of central fat redistribution and the development of insulin resistance (IR) [22, 23]. The observed older age, elevated TG levels, and higher TyG index in the plaque group are consistent with the features of “postmenopausal metabolic syndrome” [24] and the natural progression of atherosclerosis with aging. The higher prevalence of chronic angina pectoris further corroborates the link between carotid plaques, as a marker of systemic atherosclerosis, and coronary artery disease.

A notable finding of this study is the distinct pattern of association observed: the TyG index, a surrogate marker of insulin resistance, was independently associated with carotid plaques, while its derivatives that integrate obesity measures (TyG-BMI, TyG-WC, TyG-WHtR) failed to show significant associations. This finding appears counterintuitive at first glance, given the established role of obesity as a cornerstone of IR and CVD. However, within the specific context of postmenopause, a plausible explanation emerges. Weight gain, increased BMI, and WC enlargement in postmenopausal women are largely consequences of estrogen withdrawal, characterized by a shift of fat deposition from subcutaneous to visceral compartments [25, 26]. This implies that obesity, particularly central obesity, is highly prevalent in this population, potentially reducing its discriminative power as a risk factor. When these frequently collinear and less variable obesity metrics (BMI, WC) are multiplied with the TyG index, they may not enhance the signal but rather introduce noise, diluting the core pathophysiological signal related to IR that the TyG index aims to capture. In essence, for postmenopausal women, IR may be a more proximal driver of atherosclerosis than obesity per se. Therefore, the primary biological interpretation of our results is that the association between the TyG index and carotid plaques strongly suggests an underlying link between the IR state it reflects and early atherosclerosis. IR can accelerate atherogenesis through multiple mechanisms: inducing endothelial dysfunction and reducing nitric oxide bioavailability; promoting lipid accumulation and oxidative modification within the arterial wall; activating pro-inflammatory signaling pathways (e.g., NF-κB), leading to a chronic low-grade inflammatory state; and disrupting the coagulation-fibrinolysis balance, creating a pro-thrombotic milieu [27–29]. Our findings suggest that the metabolic dysregulation, marked by the TyG index and likely triggered by the dramatic hormonal shift, is a key intermediary in the pathogenesis of atherosclerosis in postmenopausal women.

The results of the subgroup analyses warrant careful interpretation. Although the point estimates for the TyG-plaque association were consistently positive across subgroups defined by diabetes status, obesity status, and menopause age, and interaction tests were non-significant, the associations lacked statistical significance within some subgroups. This phenomenon most likely reflects reduced statistical power due to smaller sample sizes in the subgroups rather than genuine effect heterogeneity. The non-significant interaction terms suggest that the association pattern between the TyG index and plaques may be similar across patients with different characteristics, which supports the robustness of the primary findings. Nevertheless, it also indicates that the predictive ability of the TyG index as a standalone measure might be modulated by various factors, requiring validation in larger studies, especially for extreme subgroups.

Despite the independent association observed, the limited predictive efficacy of the TyG index (AUC = 0.593) is another critical point for discussion. An AUC of this magnitude, while statistically significant, indicates that the TyG index alone has limited discriminative accuracy for identifying individuals with plaques in a clinical setting and is unlikely to be sufficient as a standalone diagnostic tool. However, its clinical utility may lie not in definitive diagnosis, but in initial risk stratification. Given its properties as a simple, inexpensive, and readily calculable measure derived from routine blood tests, the TyG index could serve as a useful and cost-effective ‘red flag’ in primary care or large-scale community screenings. A high TyG score in a postmenopausal woman could prompt clinicians to initiate a more detailed cardiovascular risk assessment. The limited predictive power of a single metric like the TyG index itself underscores that carotid atherosclerosis in postmenopausal women is a highly complex, multifactorial disease [30, 31]. The metabolic dysfunction reflected by the TyG index is an important contributor, but far from the sole determinant. Other significant mechanisms include: (1) Direct vascular effects of estrogen deficiency: Estrogen itself possesses vasodilatory, anti-inflammatory, antioxidant, and endothelial-protective properties, and its withdrawal directly impairs vascular homeostasis [32, 33]; (2) Inherent vascular aging associated with advancing age: including increased arterial stiffness and functional decline of endothelial progenitor cells [34, 35]; (3) Hemodynamic alterations: Abnormal blood flow patterns, such as those characterized by reduced carotid artery flow velocity as observed in conditions like coronary slow flow, may contribute to atherogenesis by altering shear stress on the vessel wall. (4) Unmeasured inflammatory status: Elevated levels of inflammatory cytokines such as interleukin-6 (IL-6) and high-sensitivity C-reactive protein (hs-CRP) [36, 37]; (5) Other metabolic disturbances: Factors like gut microbiota alterations and vitamin D deficiency may also play a role [38, 39]. Consequently, while the TyG index serves as a convenient marker for identifying individuals with metabolic dysregulation and associated cardiovascular risk, it is not sufficient as a comprehensive standalone predictive tool.

In summary, our findings carry important clinical implications. For cardiovascular risk management in postmenopausal women, clinicians should look beyond simple weight or BMI increases and be more vigilant in screening for the underlying metabolic dysregulation. The TyG index, being easily calculable and inexpensive, is well-suited for identifying at-risk individuals in primary care settings and large-scale screenings. Early intervention through intensified lifestyle modifications or, if necessary, pharmacotherapy to ameliorate this metabolic state, may provide a critical window of opportunity to slow atherosclerotic progression and achieve primary prevention of CVD in this population.

This study has several limitations. First, the cross-sectional design precludes the establishment of causality between the TyG index and carotid plaques. Although it is biologically more plausible that insulin resistance promotes atherosclerosis, the possibility of reverse causation cannot be entirely ruled out; underlying subclinical atherosclerotic processes might contribute to a systemic inflammatory state that could secondarily influence metabolic parameters. Second, while the TyG index is a widely used surrogate marker for insulin resistance, it is not the gold standard (e.g., the hyperinsulinemic-euglycemic clamp). Future studies incorporating measures like HOMA-IR could provide further validation. Third, we lacked data on sex hormone levels, detailed body composition analysis (e.g., visceral fat area), and other inflammatory biomarkers, preventing direct adjustment or mediation analysis to precisely elucidate the complete “hormones-body fat distribution-IR-vascular disease” pathway. Finally, the single-center design and relatively limited sample size may limit the generalizability of the findings, and the subgroup analyses might have been underpowered.

In conclusion, this study demonstrates that in postmenopausal women, the TyG index is independently associated with carotid artery plaques, whereas its obesity-combined derivatives are not. This key finding suggests that the underlying insulin resistance captured by the TyG index is a more relevant metabolic driver of atherosclerosis in this population than general or central adiposity per se. Consequently, our results support the potential utility of the TyG index as a simple, cost-effective tool for initial risk stratification, which could help identify high-risk postmenopausal women who may benefit from more intensive lifestyle interventions or targeted vascular screening. Future prospective studies are warranted to confirm its value in predicting clinical cardiovascular events.

## Data Availability

The datasets generated and analyzed during this study are not publicly available to protect patient confidentiality, in compliance with the institutional data protection policy. Anonymized data may be made available from the corresponding author upon submission of a methodologically sound proposal and subject to approval by the institutional review board.
